# The Effect of Water and Confinement on Self-Assembly of Imidazolium Based Ionic Liquids at Mica Interfaces

**DOI:** 10.1038/srep30058

**Published:** 2016-07-25

**Authors:** H.-W. Cheng, J.-N. Dienemann, P. Stock, C. Merola, Y.-J. Chen, M. Valtiner

**Affiliations:** 1 Department for Interface Chemistry and Surface Engineering, Max-Planck-Institute f. Eisenforschung GmbH, D-40213 Düsseldorf, Germany; 2 Technische Universität Bergakademie Freiberg, Physikalische Chemie II, D-09599 Freiberg, Germany

## Abstract

Tuning chemical structure and molecular layering of ionic liquids (IL) at solid interfaces offers leverage to tailor performance of ILs in applications such as super-capacitors, catalysis or lubrication. Recent experimental interpretations suggest that ILs containing cations with long hydrophobic tails form well-ordered bilayers at interfaces. Here we demonstrate that interfacial bilayer formation is not an intrinsic quality of hydrophobic ILs. In contrast, bilayer formation is triggered by boundary conditions including confinement, surface charging and humidity present in the IL. Therefore, we performed force versus distance profiles using atomic force microscopy and the surface forces apparatus. Our results support models of disperse low-density bilayer formation in confined situations, at high surface charging and/or in the presence of water. Conversely, interfacial structuring of long-chain ILs in dry environments and at low surface charging is disordered and dominated by bulk structuring. Our results demonstrate that boundary conditions such as charging, confinement and *doping* by impurities have decisive influence on structure formation of ILs at interfaces. As such, these results have important implications for understanding the behavior of solid/IL interfaces as they significantly extend previous interpretations.

Molecular structuring of ionic liquids (IL) at solid interfaces, and its relation to the chemical structure of the ions used, is a key aspect that allows tuning the performance of applications of ILs in batteries, super-capacitors, heterogeneous catalysis or lubrication^1^,^2^,^3^,^4^,^5^. IL structures at solid/IL interfaces strongly depend on both, the interfacial chemistry, as well as the intrinsic bulk properties of different ionic liquids. A detailed understanding of competing factors that govern interfacial layering including ion/ion, ion/surface interactions and their modulation by variation of the chemical structure are essential knowledge for the targeted tuning of applications of ILs^2^,^3^,^6^,^7^,^8^.

Recent diffraction studies at highly charged dielectric interfaces suggest, that a charge-stacking mechanism with stacked layers of oppositely charged ions controls charge compensation in the first layers of solid/IL interfaces^9^. Similarly, force probe experiments under potential control support the charge-stacking model at high surface charge and low water content in electrochemical systems^10^,^11^. On the other hand at low surface charging around the potential of zero charge no pronounced layering can be observed and bulk IL structure dominates the interface on mica^12^,^13^. Force probe experiments in dry ILs also demonstrated that an outer diffuse layer compensates remaining charges that cannot be compensated within the compact inner double layer due to crowding and finite size effects^14^. Recent diffraction studies^15^ and AFM force probe experiments^12^ indicate that crowding effects or polycrystalline surface structures may often also results in mixed surface bound layers of anions and cations on surfaces if a statistical average is analyzed. However, recent controversy and around electric double layer models of ILs demonstrates that interfacial structuring at solid/IL interfaces is still not fully understood^16^,^17^.

In particular, the suspected role of organic impurities in ILs^18^ and the role of water content^12^,^19^ were recently identified as main source for inconsistent data and interpretations of layering at interfaces in the literature. Specifically, complementary research by H.-W. Cheng *et al.*^12^ and by X. Gong *et al.*^19^ revealed that water has a decisive influence on interfacial structuring of 1-Ethyl-3-methylimidazolium-bis(trifluorosulfonyl)imide ([C_2_min][Tf_2_N]) on electrified gold surfaces as well as on charged mica surfaces. Also, at low surface charge on mica the stacking model is not applicable in water-free [C_2_min][Tf_2_N]^12^. Recent solution phase infrared spectroscopy studies also suggest that water molecules interact preferentially with the hydrogen atoms from imidazolium rings and the fluorine atoms from [Tf_2_N] to form specific bonds^20^. Also, recent MD simulations suggest that water impurities in imidazolium-based IL system will influence the bulk^21^ and hence the interfacial structuring drastically.

Moreover, it has been shown that solvophobic interactions, i.e. specific depletion of cations around anions and vice versa, lead to sponge like nano-structuring of bulk ILs^22^,^23^,^24^,^25^. Due to competing interactions between tails, head group and anions formation of nanostructured domains is expected in bulk ILs with increasing hydrophobic volume fractions compared to charged fractions. This led to the interpretation that ILs that contain cations with long hydrophobic tails form structures that compare to lipid bilayers at solid/IL interfaces^26^.

How effectively solvophobic interactions may order ions at solid/IL interfaces can even more so depend on impurities present in ILs. Yet experimentalist largely ignored this aspect. In particular, increased water concentration at the interface and around charged groups may trigger hydrophobic association in ILs and at interfaces^21^. As such, how boundary conditions, impurities and environmental conditions influence the subtle balance between ion/ion, ion/surface may have a substantial and decisive impact on interfacial structuring of ILs as a function of the chemical nature of the ions used.

## Results and Discussion

**Figure 1:**
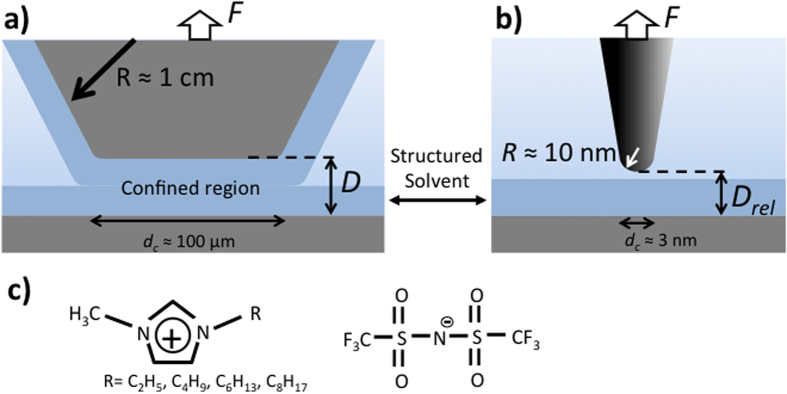
Schematic of force probe measurements between two apposing surfaces across ionic liquids under macroscopically confined and nano-confined geometries by using (**a)** surface force apparatus and (**b)** atomic force microscopy, respectively. (**c)** The imidazolium based ionic liquids with different length of hydrophobic unit were used in this study (*cf.* text for details).

As shown in Fig. 1a,b, here we measured atomic force microscopy (AFM) based 2D force versus distance characteristics (2D-FD) at extended mica surfaces, and we recorded ensuing force versus distance characteristics of ILs confined between two apposing macroscopic mica surfaces using the surface forces apparatus (SFA) as a function of the water content in a series of differently hydrophobic ILs. In particular, we studied the relation between water concentration and interfacial structuring on mica (001) surfaces of a series imidazolium based ionic liquids with increasing hydrophobic tail length on the cations that are shown in Fig. 1c.

Furthermore, we utilized Karl-Fischer (KF) titration to characterize water content, as well as uptake and release dynamics of water in the ILs used. The results of the KF-titration in Figure S1 indicate that the water release/uptake kinetics in all these system is quite similar and does not depend on the degree of hydrophobicity of the cation. Also, KF-titration behavior suggests that the maximum water capacity of ILs is closely related to the ion pair density, with only slight deviations from linearity due to the increasing length of the hydrocarbon chain. As such, a weak correlation of the chain length and the maximum water content indicates strong association of water with the hydrophilic domains. This behavior agrees well with experiments and simulations^20^,^21^ indicating that water molecules preferentially form hydrogen bonds with the imidazolium ring and the [Tf_2_N] anion. However, at interfaces water and ion concentrations may show significant depletion or excess of either species (ions or water) compared to bulk concentrations.

**Figure 2:**
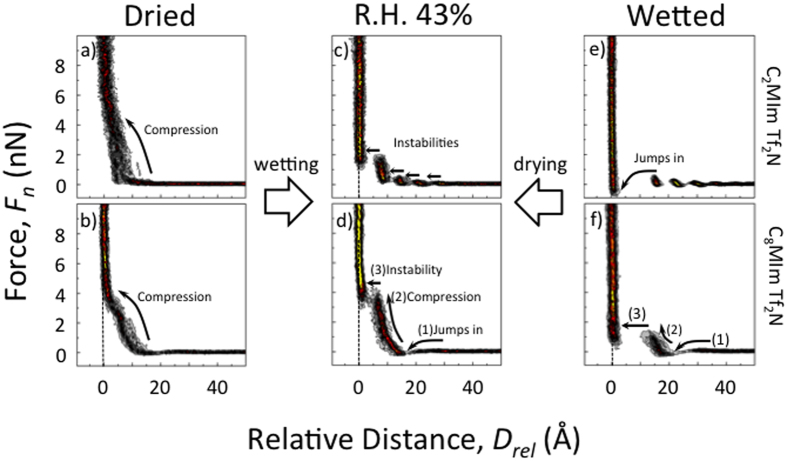
AFM based 2-dimension-force distance (2D-FD) spectroscopy of (**a,c,e)** [C_2_min][Tf_2_N] and (**b,d,f)** [C_8_min][Tf_2_N] at various water concentrations as indicated. All 2D-FD plots were generated from 40 random selected AFM force distance measurements (*cf.* text for details).

Figure 2 shows 2D-FD AFM force spectroscopy at extended mica surfaces immersed in [C_2_MIm][Tf_2_N] and [C_8_MIm][Tf_2_N] as a function of the water concentration, respectively. Force versus distance characteristics of all ILs used are shown in the supporting information (Figure S2). A gradual shift of the force distance characteristics was observed between these two extremes. Here, we only plot the two extreme cases. Several interesting aspects can be directly observed in these data.

First, independent of the hydrocarbon chain length of the cation no strongly pronounced liquid structuring was observed on mica surfaces in dry conditions (see Fig. 2a,b). In contrast for both [C_2_MIm][Tf_2_N] and [C_8_MIm][Tf_2_N], a continuous force profile with an exponential repulsion kicking in at D ~ 12–14 Å indicates a thin adsorbed, yet unordered layer of IL molecules at the interface. Such force versus distance characteristics typically indicate thermally disordered layering of ions at the interfaces. In particular, the data for [C_2_MIm][Tf_2_N] compares well with our previous measurements^12^ and data by Sakai *et al.*^27^ indicating a final compression layer at D ~ 5 Å, and weak structuring at larger distances. Compared to Sakai *et al.*^27^ we rely on a statistical analysis of data measured multiple times and over different areas as suggested by Black *et al.*^10^,^11^. In the statistical analysis we find that structured layering at the outer layers in dry IL is statistically not pronounced (see Figure S3), while it appears pronounced in individual force distance measurements. We also find no qualitative change of the characteristics due to variation of approach speeds in the range from 20–200 nm/s. Yet, at slower compression speed the outer diffuse layer becomes statistically more pronounced. This behavior indicates a generally very unstructured and not highly ordered IL/mica interface in dry ionic liquids. Based on additional XPS analysis (Supporting Figure S4), the surface adsorbed liquid layer statistically contained a relative higher anion concentration (from > 50 XPS spectra), which agrees well with an anion over-adsorption model suggested by recent work on silica-based systems^28^,^29^.

Interestingly, and as can be seen in Fig. 2b, also the repulsion observed in [C_8_MIm][Tf_2_N], that kicks in at D ~ 12–14 Å, followed by the rapid approach from D ~ 5 Å into contact at D = 0, supports an electric double layer model with an inner adsorbed layer of anions and a disordered outer layer of cations over about 12–14 Å. Hence, the force distance profiles in dried IL indicate an anion over adsorption, which is compensated by a disordered cation layer. In contrast to previous work^26^,^30^ our data suggests that dried imidazolium based ionic liquids show very weak or no interfacial structuring at mica surfaces, irrespective of the chain length of the cations used. In particular, Fig. 2b shows no indications that longer chain cations (here [C_8_MIm]^+^) form bilayers at an extended interface.

However, and as can also be seen in Fig. 2, once water was introduced into the IL/mica system, the interfacial structure of all ionic liquids started to develop strongly pronounced layered structures as indicated by multiple observed instabilities. In particular, after the systems equilibrated with 43% relative humidity surrounding as shown in Fig. 2c,d, the gradual compression mechanism changed to a discontinuous force profile with apparent instabilities in the profiles. As shown previously^12^, during equilibration with relative humidity force profiles gradually show more and more layering over time, which agrees well with the measured water uptake dynamics shown in Figure S1.

In agreement with work by Christenson and Horn with hydrophobic liquids water, likely adsorbs at the mica interface^31^,^32^. In silanes 240 ppm of water directly adsorbed at the interface, but did not lead to charging of the mica surface^31^. Hence, in organic solvents such as silanes^31^,^32^ it became clear that water directly goes to the interface, while charging was possibly not favorable due to small solubility of hydrated K^+^ in silanes and/or kinetic limitations. In ionic liquids a subtle balance between water at the interface, solubility of hydrated ions, and water in the bulk IL must be expected. As previously argued, the humidity-dependent increase of layering is a strong indication that water induces increased surface charging^12^,^19^ by potassium dissolution from mica surfaces. Apparently, in the hydrophobic ILs used in this work, a concentration in the range of ~1000 ppm (equilibrium with ambient humidity) is sufficient to charge the mica surface.

Consequently cation adsorption dominates the charge compensation mechanism in the inner double layer in humid systems. Compared to the dried ionic liquid system in Fig. 2b, four strongly pronounced instabilities were observed for [C_2_MIm][Tf_2_N] at r.h. of 43% shown in Fig. 2c. This behavior indicates strongly adsorbed ion layers with a thickness of ~6.6–7.7 Å, which correlates with the expected ion-pair diameter and a stacked ion layering^33^.

Yet, only two instabilities were observed in humid [C_8_MIm][Tf_2_N] as shown in Fig. 2d. As indicated, (1) an attractive jump-in over 16 Å was observed at D ~ 28 Å from the hard wall, and (2) at smaller separations D < 15 Å the normal force continuously increased to around 4 nN over a steep 5 Å compression regime, (3) followed by an instability with 7 Å jump-in to D = 0. This suggests charge compensation by a structured and adsorbed layer of oriented cations at D < 15 Å, and an outer depletion layer that leads to measurable depletion attraction at D > 15 Å, as indicated by the jump in. The observed depletion attraction, which cannot be explained by a Van der Waals attraction, clearly indicates that no bilayers formed in dry [C_8_MIm][Tf_2_N]. Instead, the positively charged immidazolium ring may face the negatively charged surface and the tail may face the bulk IL, which compares to a self-assembled monolayer structure. The observed compression behavior with the instability at ~7 Å indicates a dilute monolayer with a liquid-phase behavior that can be compressed considerably over 5 Å before the layer is pushed out.

Figure 2e,f show the AFM results from water saturated [C_2_MIm][Tf_2_N] and [C_8_MIm][Tf_2_N], respectively. In fully water-saturated conditions, both systems exhibit strongly pronounced interfacial structuring. In wet [C_2_MIm][Tf_2_N] shown Fig. 2e, layering extends to D ~ 40 Å with similar instabilities of 6–7 Å compared to the humid case shown in Fig. 2c. Again, the presence of water leads to potassium ions hydration and dissolution into the liquid phase and high surface charging and consequently strongly structured ion layering that extends over several molecular ion layers^12^,^19^. However, in fully saturated conditions, a final instability results in a 15 Å jump-in to D = 0. This suggests that adsorption of a nanoscopic water layer at the mica surface may trigger the formation of a water-capillary between the mica and the nanoscopic AFM tip surfaces, which leads to a long-range attractive force contribution and complete disappearance of layering in AFM signals. This capillary attraction between tip and surface also reduces the force required to penetrate the IL layers and facilitates a jump into contact at larger distances. This correlates well with observed lubrication behavior of humid ILs, where breakdowns of stable sliding layers where observed in humidified systems^4^,^5^. Also, previous work by Sekai *et al.*^27^ indicates very similar results for very wet [C_2_MIm][Tf_2_N] with increasing distances of the capillary instability and reduced forces required to penetrate IL layers, if the water concentrations increase from 12000 ppm to 20000 ppm. As can be clearly seen in Fig. 2, we also find increasing structuring when going from very wet, to humidified ILs, while structuring almost entirely disappears, if the ILs used are dry with the surface remaining uncharged.

Qualitatively similar results were observed for the [C_8_MIm][Tf_2_N] system, and are shown in Fig. 2f. However, with the increasingly longer hydrocarbon side chains on the cation structuring in the fully water saturated IL extends over fewer ordered layers, compared to shorter chain lengths. The observed instability at D ~ 11 Å in the wet [C_8_MIm][Tf_2_N] system, which is on the order of the length of the cation, indicates the presence of a more densely absorbed cation layer with its hydrophobic side chains pointing into the water rich liquid IL phase. Compared to the behavior observed at 43% r.h. there is no pronounced compression behavior at D < 11 Å. Instead, a jump into contact at D = 0 at lower forces was observed. This suggests a higher mobility of the adsorbed interfacial cation layer on water saturated mica surfaces.

In addition, the repulsive compression at 11 Å < D < 20 Å indicates the formation of a second outer layer that adsorbs onto the inner strongly adsorbed layer. Since this compression extends over about 11 Å as well, the data suggests that cations adsorb on top of the surface absorbed cation layer through hydrophobic interactions in these water-saturated systems. This is in excellent qualitative agreement with bilayer model proposed by Perkin *et al.*^26^. However, the outer second layer consists of a much less dense cation layer with a strong tendency to disorder into the bulk IL. In addition, the jump-in at D ~ 30 Å again indicates a depletion attraction at the interface. As such this data indicates water-induced bilayer formation with a dense inner and a dilute liquid-phase outer layer on extended mica surfaces in water saturated hydrophobic ILs. Compared to lipid bilayers this behavior compared well to a liquid phase bilayer with a very dilute outer layer.

We additionally also studied how confinement over large areas during interaction influences the structuring of IL with increasing carbon chain length of the cation as a function of the water concentration. Unlike AFM experiments with small probing area, the surface forces apparatus (SFA) is able to approach two individual extended mica surfaces to confine the ionic liquid in between over contact areas of ~1000 μm^2^ (see again Fig. 1).

**Figure 3:**
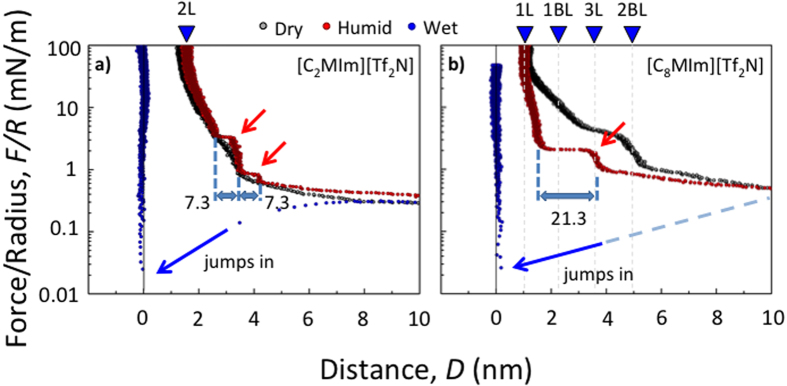
SFA force versus distance characteristics of (**a)** [C_2_min][Tf_2_N] and (**b)** [C_8_min][Tf_2_N] during compression between two mica surfaces in dry (black), with 43% humidity equilibrated (red), and water saturated (blue) ILs.

**Figure 4:**
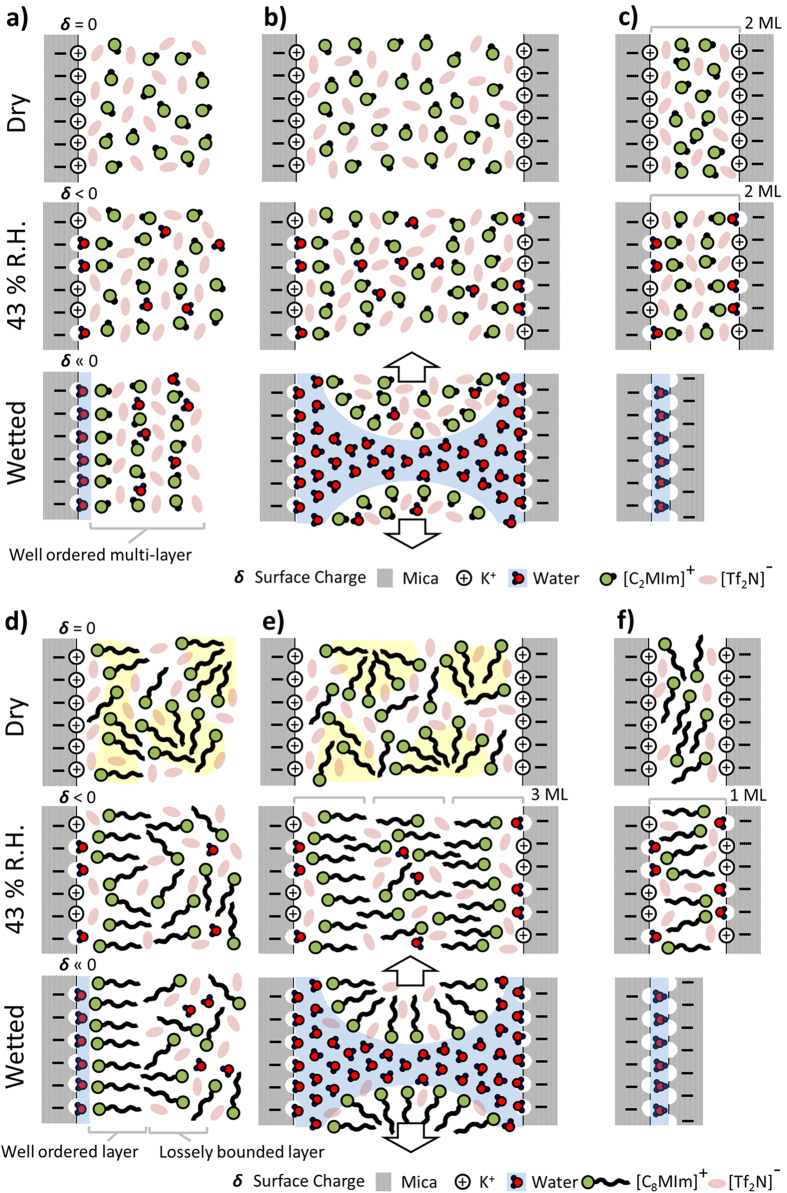
Schematic interpretation of the interfacial structures of [C_2_MIm][Tf_2_N] and [C_8_MIm] [Tf_2_N] on mica surface with various water contents under (**a,d)** no confinement (AFM), (**b,e)** confinement at distances of a few molecular diameters and (**c,f)** hard compression in confinement with SFA.

Figure 3 shows ensuing SFA force-distance characteristics of a) [C_2_MIm][Tf_2_N] and b) [C_8_MIm][Tf_2_N] under dry (black), 43% humidity (red) and wet (blue) conditions. Force versus distance characteristics of all ILs used are shown in the supporting information (Figure S5). In general, most of the results displayed in Fig. 3 agree qualitatively well with the observation in nanoscopic AFM studies. Firstly, absence of instabilities and hence no extensive layering was observed in dried ionic liquids, irrespective of the carbon chain length of the cation. Secondly, pronounced layering, with instabilities in the force profiles, only appears in the presence of traces of water. Thirdly, and as schematically shown for the wet case in Fig. 4(a–c), strong capillary attractions are observed in wet, fully water-saturated, conditions where the apposing surfaces jump into contact at D = 0 from large distances D > 4–10 nm, irrespective of the used IL.

The following interesting aspects specifically differ and/or compare well in SFA and AFM data of the differently hydrophobic ILs used. First, and as can be seen in Fig. 3a, the range of the instability and the jump-in events in [C_2_MIm][Tf_2_N] agrees well with the value recorded in AFM studies of 6.6–7.7 Å, corresponding to an ion pair diameter. Hence, in both techniques an ion-pair push-out dominates the compression mechanism during increasing confinement. As such, both techniques seem to reflect similar information about the structuring, which is possibly based on a stacked pairwise layering of cations and anions. Second, due to the different contact geometry in SFA experiments the ion layers in dry and the humid case (43% *r.h.*) cannot squeeze out of confinement, due to the significantly lower pressures that can be reached in SFA at maximum. Under the highest possible compression where the distance does not change upon increase of the force, the separation distance between two mica surfaces in an SFA experiment is about 15 Å, which indicates that an equivalent of about two ion pair layers are confined between two mica sheets at the given pressure in [C_2_MIm][Tf_2_N] as shown in Fig. 4(a–c). This data agrees well with SFA data of most other groups, and indicates that IL remains between the two opposing surfaces in an SFA experiment, if the system is dry or if no shear forces are applied^4^,^5^.

Second, and in contrast to AFM data Fig. 3b shows a completely different force distance profile for [C_8_MIm][Tf_2_N] compared to AFM in both 43% relative humidity and the dry case. In the humidified system at 43% r.h. first a long-range repulsion followed by a stable layer at D ~ 38 Å was observed for the [C_8_MIm][Tf_2_N] system. Upon further compression a 20 Å jump-in to D ~ 15 Å, and an additional compacting to a separation distance of D ~ 11 Å at maximum compression was measured. This final compression distance is in good agreement with a hypothetical layer thickness of one monolayer of strongly adsorbed [C_8_MIm]^+^.

This data suggest that two opposing cation bilayers can apparently compress into one cation monolayer that compensated for the charge of both surfaces. The stable layer at D ~ 38 Å indicates a fusion of the opposing bilayers into a stacked 3-layer system, with two densely adsorbed layers at either of the opposing surfaces and an intermediate intercalated layer of the two dilute outer layers. The observed rapid compression over ∆D ~ 22 Å upon further compression correlates well with a squeeze-out mechanism of the proposed weakly bound cation layers which are adsorbed onto a more densely adsorbed inner layer. In extension of previous work^26^,^30^ our data further indicates that the two dilute outer layers can be compressed into one intercalated layer, followed by an instability occurring at a distance D ~ 35 Å. Upon further confinement, the surfaces rapidly approach to D ~ 20 Å, which is typical for a hemi-fusion, with a contact at the distance of two opposing monolayers. Subsequently, the interfacial hemi-fused layers compress into a distance that corresponds to one adsorbed monolayer of cations at D ~ 11 Å. Hence, and as schematically shown for the humid case in Fig. 4(d–f) both AFM and SFA data indicate water-induced bilayer formation with a denser inner and a very dilute liquid-phase outer layer on extended mica surfaces in water saturated hydrophobic ILs.

Interestingly, the dry [C_8_MIm][Tf_2_N] system also shows considerable deviations from the behavior observed in the AFM experiments. In particular, and as can be seen in Fig. 3b, the force profile shows a long range force repulsion followed by two undulations in the force distance characteristics. The possible implications of weak long-range repulsion at D > 5 nm, have been discussed in detail elsewhere^14^,^34^. Importantly, these undulations appear at distances that do not correlate with the expected dimensions of interacting strongly ordered bilayer like structures (i.e. solid like bilayers), which are marked as 1BL and 2BL in the plot in Fig. 3b. In particular, the measured data shows no sharp increase of the force distance profile. Quite in contrast, a rather smooth and long ranged continuous compression with two changes of the slope was observed. The first slope change occurs at D ~ 50 Å, which compares to the thickness of two bilayers. The second instability occurs at D ~ 30 Å, which correlates with a nominal thickness of 3 monolayers of cations.

As schematically shown for the dry situation in Fig. 4(d–f), considering the weak structuring measured on the extended surfaces using AFM, this data suggests that the observed behavior reflects the compression of IL bulk nanostructures structures into a single layer with over adsorbed anions and unordered cations, under influence of increasing confinement, rather than compression of structures adsorbed at the surface. In particular, this compression mechanism compares well to e.g. structural compression mechanism that was observed for lipid vesicles adsorbed from aqueous solutions. Hence, we propose that dry ILs containing cations with long carbon chains reflect their internal structuring bulk liquid into measurements under confinement. No evidence for a stable and strong interfacial bilayer formation was observed in dry conditions and on uncharged mica surfaces, which extends current IL interface models^26^,^30^. The observed characteristics of compressive undulations are within the range of the dimensions of bulk structures that where observed in simulation experiments^21^,^22^,^23^,^24^,^25^,^33^,^35^.

## Conclusions

In conclusion, the presence of small amounts of water in the system seems necessary to charge the mica surface. Our data suggests that water causes pronounced ion layering and triggers interfacial structure formation in all ILs studied here. The results in [C_8_MIm][Tf_2_N] suggest that bilayer like interfacial structures only occur, if sufficient water is present in the system, and if the respective surfaces are highly charged and/or confined. In contrast, extended surfaces in humid and wet systems only show formation of a monolayer of adsorbed cations, with a dilute fluid-like second outer layer. Conversely, layering of dry ILs at uncharged dielectric surfaces is diffuse and unstructured, irrespective of the chain length of the cation. Based on XPS data in dry ILs it is likely that anions over adsorb on potassium terminated mica surfaces, and a diffuse outer EDL forms. As such, dry ILs exhibit no intrinsic driving force to form bilayer like surface structures at mica surfaces, leading to relatively unordered interfaces with structuring that does not deviate much from bulk structuring. Clearly, our data suggests that water and possibly other small molecules may have a very strong impact on interfacial layering of ILs, offering also tool for tailoring IL interfaces in future applications. As such, our data and the approach have important implications for fields where interfacial layering of ionic liquids is a key aspect to tailor functionality including friction and lubrication, catalysis, as well as energy applications of IL.

## Methods and Materials

### Chemicals

1-Ethyl-3-methylimidazolium bis(trifluoromethylsulfonyl)imide ([C_2_MIm][Tf_2_N], purity: ≥98% NMR), 1-Butyl-3-methylimidazolium bis(trifluoromethylsulfonyl)imide ([C_4_MIm][Tf_2_N], purity: ≥98% HPLC) and 1-Hexyl-3-methylimidazolium bis(trifluoromethylsulfonyl)imide ([C_6_MIm][Tf_2_N], purity: ≥98% HPLC) were purchased from Sigma-Aldrich. 1-Octyl-3-methylimidazolium bis(trifluoromethylsulfonyl)imide ([C_8_MIm][Tf_2_N], purity: ≥99.9%) was purchased from Solvionic. For volumetric Karl-Fischer titration (TitraLab KF1000), HYDRANAL^®^-Titrant 5 with a water equivalent of 4.95~5.05 mg/mL and HYDRANAL^®^-solvent E (ethanol based) were purchased from Sigma-Aldrich.

### Sample Preparations

In order to remove the residual water to obtain dry samples, all the ionic liquids were dried in a vacuum oven at 80 °C overnight. The oven was then vented with dry argon gas. Karl-Fischer titration showed that the remaining water concentrations in ionic liquid were around 200 ppm after transfer procedures into experiments. For the humid sample preparation, the ionic liquids were mixed with Milli-Q water (organic impurity ~2 ppb) in glass container and mixed for 30 seconds. After mixing, samples were equilibrated around 30 minutes until the phase boundary between ionic liquids and water became sharp and clear.

### Karl-Fischer Titration

A dried syringe was used to transfer fully dried/humidified ionic liquids to glass beakers for KF-titration and for transfer into dried experimental chambers and cells.

### Atomic Force Microscope (AFM)

All AFM experiments were carried out with Nanowizard 1 (JPK Instruments, Germany) with a 15 mm diameter home-build fluid cell that can be flushed with a continuous stream of dry Argon, to prevent ingress of humid atmospheres. All measurements were performed with silica-terminated silicon AFM tips (Budget Sensor, CONT, typical tip radius R_tip_ ~5–10 nm) with a spring constant of ~0.06 N/m as determined by the thermal noise method. AFM tips were carefully cleaned in concentrated sulfuric acid (purity: 95 ~ 98%, Sigma-Aldrich) for 60 seconds. Afterwards tips were washed with Milli-Q water and ethanol (purity: 99.8%, Sigma-Aldrich) respectively, and dried in a stream of nitrogen. In order to minimize the water concentration variation over the time, the first set of measurements were acquired within 10 minutes after the mica surface came into contact with ionic liquids. For force measurements, a 10 × 10 grid was set on a 100 μm^2^ area. 3 force measurements were acquired from each point with approach speeds of 0.2 μm/s and up to 20 nN normal force load, in order to apply high enough pressures for determination of the hard walls in AFM^36^. Given the small tip radii used (R ~ 10 nm) the applied approach speed is slow enough to suppress dynamic effects due to viscous drag^36^. In any case we also tested much slower approach speeds of 20 nm/s as and did not find any differences (see Figure S3). We want to however emphasize that inherent variations in tip radii and hence confined areas lead to quantitative differences in the measured forces, while qualitatively results are well reproducible across different tips and experiments. We do not normalize by tip radii and use different tip for each set of experiments in order to minimize cross contaminations. Results that are show for one particular IL were recorded with a single tip. Hard walls (i.e. where the force increased steeply at constant distance) were aligned and defined as D = 0. The sample surface was freshly cleaved [0001] muscovite mica (optic grade V/1). Carbon contamination and cleanliness of the surfaces was checked by angle resolved *x*-ray photospectroscopy, and showed similar amounts of contamination compared to previous literature studies (see Figure S4). 40 out of 300 random selected force curves were used for generating 2D-FD plots. 5 times 10^−11^ m and 10^−10^ N were set as binning for the x-axis and the y-axis for generating 2D-FD plots, respectively.

### Surface Force Apparatus (SFA)

Force versus distance characteristics of confined ionic liquids at ~1000 μm^2^ contact area were measured using a Surface Forces Apparatus 2000 (SurForce LLC, Santa Barbara, USA) with controlled environment (23 °C room temperature, dried/humidified Ar gas purged). In contrast to our AFM experiments, this results in a fully equilibrated system with constant water concentrations. Two apposing mica (same quality as AFM experiment) surfaces were used in a 3-layer interferometer setup to perform the experiment^37^,^38^. Prior to every experiment, the SFA chamber was first purged by dried Ar gas ~30 min to remove residual humidity. Experiments with dried ILs were performed under gentle Ar flow to maintain a constant over-pressure in the SFA chamber during the measurement. Afterwards, the front-lid of SFA chamber was opened to allow equilibration of the ionic liquid with humid air in order to mimic exposure to realistic natural environments (43% relative humidity as controlled by a humidifier in a controlled clean-room environment, Class 1) for 3 hours. Experiments at 43% relative humidity were then carried out with the front lid open to the controlled environment. Finally, humidified AR gas was purged into the chamber with the lid closed for ~1 hour to fully wet the ionic liquid for measurements. All the measurements of individual ionic liquids were done with the same contact. To minimize hydrodynamic drag during approach, extremely slow approach speeds of 3~5 Å/s were used to perform quasi-static experiments. The zero distance, where D = 0, was shifted to the hard wall recorded in wet ionic liquid systems after jump into contact. Hence, the contact position is always referenced to an intimate mica/mica contact where K^+^ ions dissolved into the bulk IL. This reference point is more intuitive in the given context, compared to using the dry contact of two mica surfaces without any IL present (which was recorded as usual).

## Additional Information

**How to cite this article**: Cheng, H.-W. *et al.* The Effect of Water and Confinement on Self-Assembly of Imidazolium Based Ionic Liquids at Mica Interface. *Sci. Rep.* **6**, 30058; doi: 10.1038/srep30058 (2016).

## Supplementary Material

Supplementary Information
