# Supplementary material for: The Effect of Water and Confinement on Self-Assembly of Imidazolium Based Ionic Liquids at Mica Interfaces
Source: Sci Rep. 2016 Jul 25;6:30058. doi: 10.1038/srep30058 (PMC4958918; doi:10.1038/srep30058)
Supplement: Supplementary Information [file srep30058-s1.pdf]

Supporting Information:

The Effect of Water and Confinement on Self-Assembly of Imidazolium Based Ionic Liquids at Mica Interface

*H.-W. Cheng, J.-N. Dienemann, P. Stock, C. Merola, Y.-J. Chen and M. Valtiner**

Department for Interface Chemistry and Surface Engineering, Max-Planck-Institute f. Eisenforschung GmbH, D-40213 Düsseldorf, Germany.

Karl Fisher Titration.

The results of the KF-titration in **Figure S1a** show both (1) the water release from fully water–saturated ionic liquids after contact with air (43% relative humidity), as well as (2) the water uptake into dry ionic liquids as a function of the varying cations. First, both wet and dry samples exponentially reached an equilibrium water concentration after ~3 hours in contact with 43% relative humidity. This indicates that the water release/uptake kinetics in all these system is quite similar and does not depend on the degree of hydrophobicity of the cation.

Second, the saturated water concentrations within ionic liquids in equilibrium with aqueous water (**Figure S1a** at t = 0) strongly depend on the IL used. In particular, [C_2min] [Tf_2N] is capable to store the highest amount of water and [C_8min] [Tf_2N] the lowest amount, after equilibration with a liquid water phase. At first glance, this supports a direct relation of the chain length of the hydrophobic tail of the cation and water uptake. However, the maximum water concentration can also be replotted against the concentration of the ion pairs as shown in **Figure S1b**, which is the relationship between the water capacity and ion pair density. **Figure S1b**, indicates a strong correlation of water and ion pair concentrations. In addition to testing the series of ILs with increasing hydrocarbon chain length, we also recorded

the maximum water content in binary mixtures of the most hydrophilic [C_2min][Tf_2N] and most hydrophobic IL [C_8min][Tf_2N], as indicated in **Figure S1b** as well. As can be seen in the figure, mixing of the two extremes results in a strong linear correlation of ion-pair density and maximum water content with a slope of 0.9 water molecules per additional ion-pair per unit volume. This behavior suggests that the maximum water capacity of ILs is closely related to the ion pair density, with only slight deviations from linearity due to the increasing length of the hydrocarbon chain.

As such, this weak correlation of the maximum water content with the hydrocarbon tail lengths indicates strong association of water with the hydrophilic domains. This behavior agrees well with experiments and simulations [1, 2] indicating that water molecules preferentially form hydrogen bonds with the imidazolium ring and the [Tf_2N] anion. However, at interfaces water and ion concentrations may show significant depletion or excess of either species (ions or water) compared to bulk concentrations.

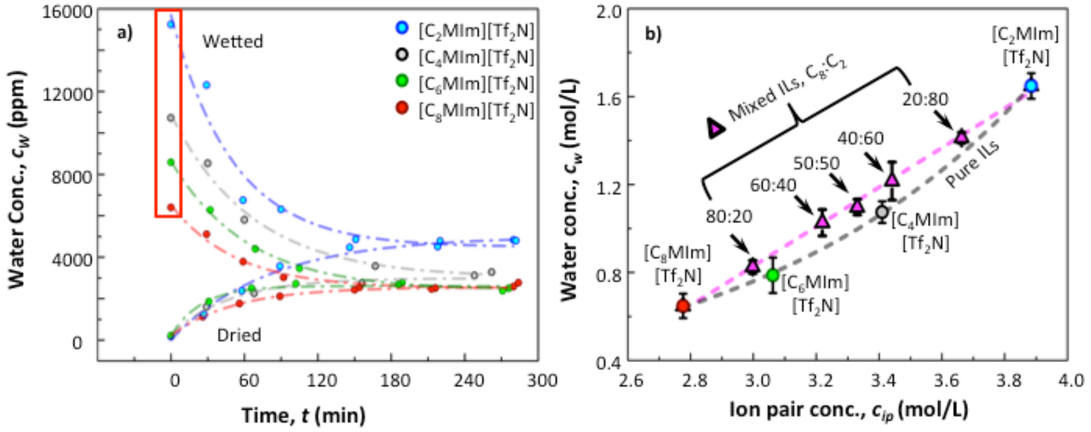

Figure S1. a) Change of the water concentration in dried and water saturated ILs during equilibration with a 43% relative humidity surrounding. b) Water concentration (mol/L) of water saturated ionic liquids and a series of binary mixtures of [C_2MIm][Tf_2N]:[C_8MIm][Tf_2N] versus the ion pair concentration (mol/L).

Table S1. Comparison of measured and published maximum water content for the homologous $C_nC_1Im\ Tf_2N$ series of ILs in unit of molar ratio $\boldsymbol{X_w}$ of water in ionic liquid.

Ionic liquid	Measured Value (X_W)	Literature Value
$C_2MIm\ Tf_2N$	0.302 ± 0.003	0.298 ± 0.009
$C_4\ MIm\ Tf_2N$	0.240 ± 0.008	0.257 ± 0.006
$C_5\ MIm\ Tf_2N$	-	0.221 ± 0.005
$C_6\ MIm\ Tf_2N$	0.204 ± 0.017	
$C_7\ MIm\ Tf_2N$	-	0.197 ± 0.004
$C_8\ MIm\ Tf_2N$	0.197 ± 0.008	
$C_9\ MIm\ Tf_2N$	-	0.174 ± 0.001

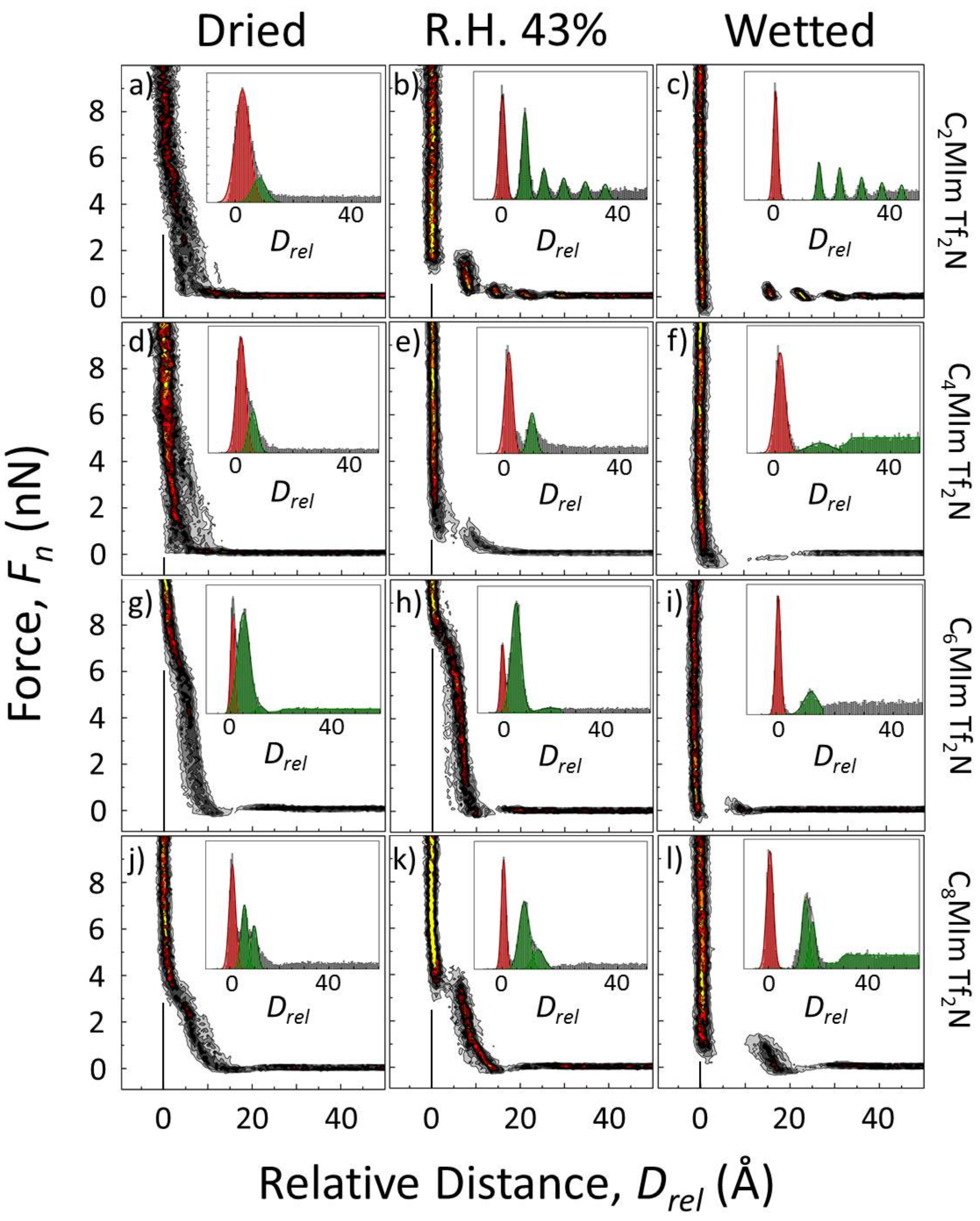

Figure S2. Full set of AFM data for series of ILs, including [C_2min][Tf_2N], [C_4min][Tf_2N], , [C_6min][Tf_2N] and [C_8min][Tf_2N] in wet, dry and humid environments, respectively. The small insets show a density plot of the observed layering. Absolute forces across the series cannot be interpreted because each set of data for a particular IL was performed with individual tips of slightly varying radius (R ~ 5-15 nm), which may result in quantitative differences up to a factor of 2-3. We refrain from using one tip in many different ILs in order to avoid cross contaminations.

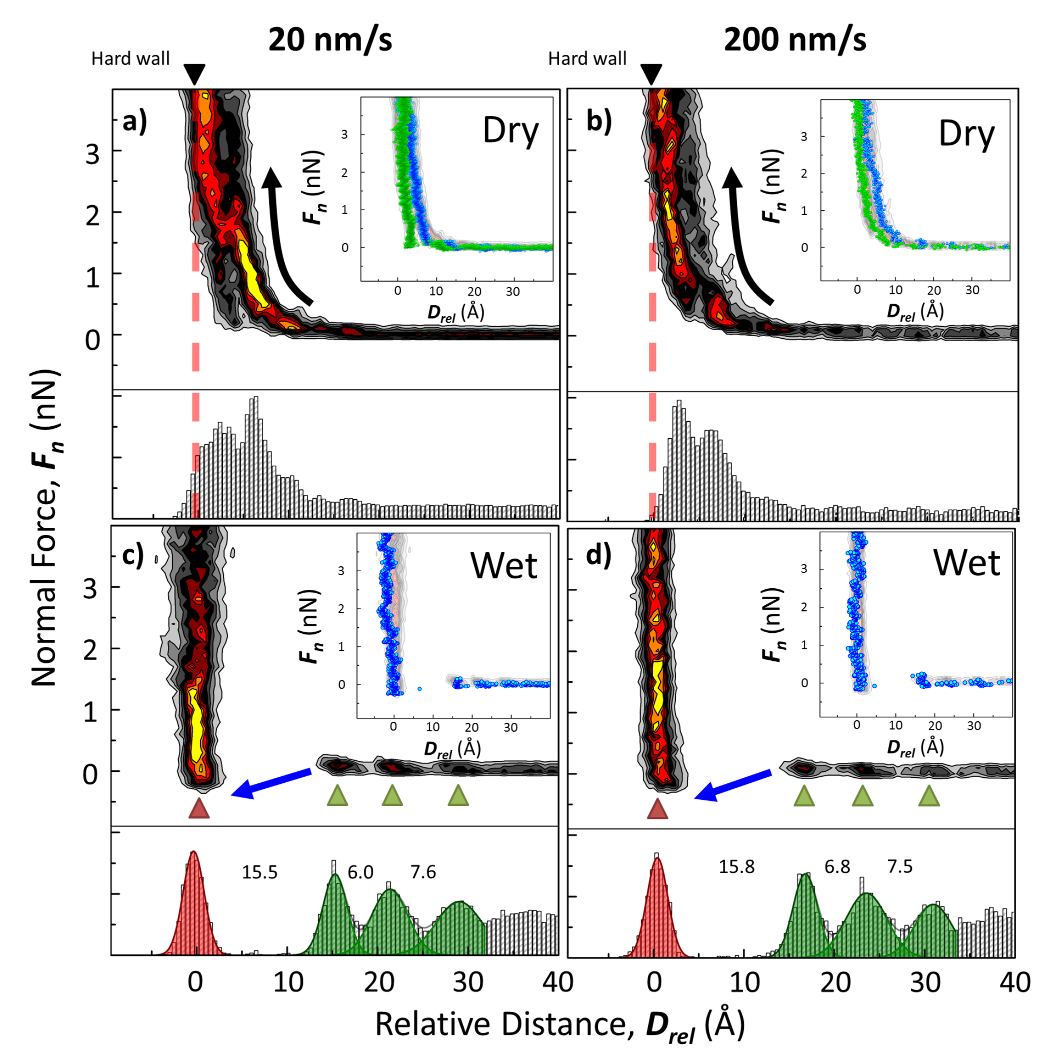

Figure S3. Comparison between slow and fast approach speeds of (a, b) 20 nm/s and (c, d) 200 nm/s, in dry and wet [C_2MIm][Tf_2N] respectively. As expected, the approach speed shows very limited effects and the results are qualitatively similar. In dry [C_2MIm][Tf_2N] events showing higher compression forces to push out diffuse layers at D > 5 Å are statistically more pronounced (see also in the histograms shown below). Clearly, in both a final layer with a 2-3 Å thickness is detectable, followed by 2-3 weak structural layers that are very diffuse in a statistical analysis. The small insets show statistical data overlaid by "typical" individual curves that indicate strong layering. As can be seen, "typical curves" are potentially misleading. In particular individual curves do not capture the statistical nature of the weak layering in the dry case, while they are representative for the overall structuring in the wet case.

Photoelectron Spectroscopy (XPS):

All XPS measurements were performed with a Quantum 2000 (Physical Electronics). All spectra were measured with the "high-power mode" of the used machine. In this mode photoelectrons are generated by continuously scanning a 100 × 100 μm^2 sized X-ray spot (100 W) over an area of 100 × 1000 μm^2 in order to minimize X-ray damage to the sample. Using a pass-energy of 26 eV, and a spectral resolution of 0.1 eV. To obtain a good signal-to-noise ratio 10 and 15 sweeps for the C 1s and N1s signal, respectively. All the binding energies are calibrated by Si 2p signal (102.16 eV for muscovite mica, see LaSurface.com database)

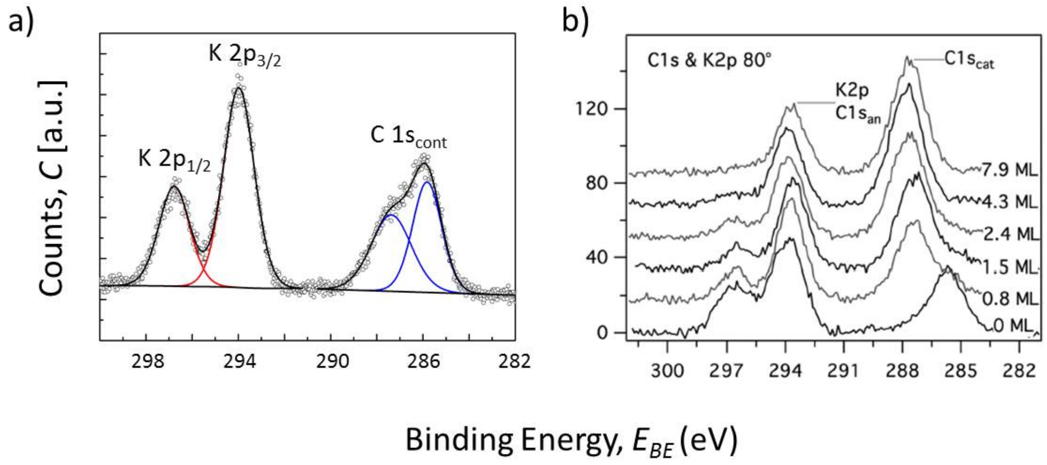

Figure S4 shows the angle-resolved X-ray photospectroscopy (ARXPS) comparison between a) measured spectrum from freshly cleaved muscovite mica used in this study and b) the reported spectrum from literature in carbon-potassium region at 80°. Comparing K and C signal intensities, it is clear that the carbon contaminations in the present study are comparable with the cleanest case reported in the literature [3]. Notice that the sources of carbon contaminations from the air or even from the mineral itself might differ case by case, and we also found higher contaminations for some cleaved micas.

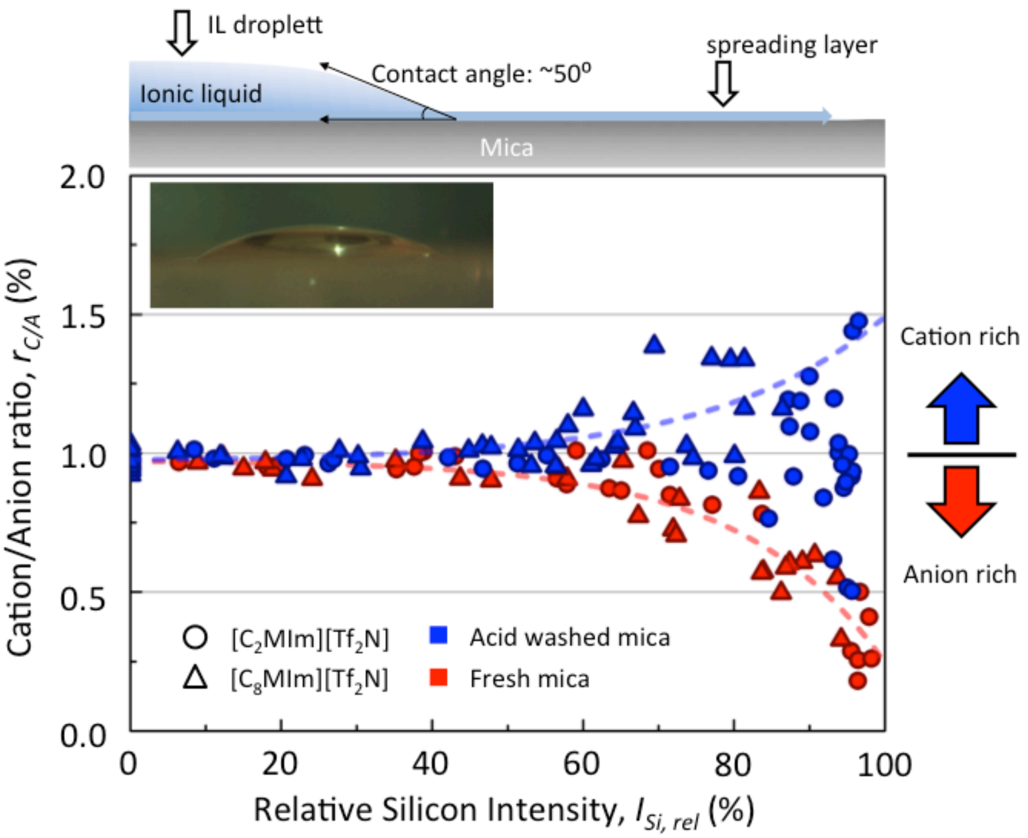

Figure S5. XPS spectra of spreading droplets of [C_2min][Tf_2N] and [C_8min][Tf_2N] on freshly cleaved and acid washed mica recorded at 0 degree detector angle. On acid washed mica the surface terminating K^+ ions where ion-exchanged with protons by immersion into an HCl solution at pH = 1. Hence, in an IL, the surface can charge via deprotonation. The small inset shows a contact angle measurement, which estimates a contact angle of ~50 degree for both ILs. Still a thin spreading layer extends much further way from the droplet, and can be observed in XPS even a few mm away from the bulk liquid droplet. To investigate the specific ion absorption on mica surface, a small drop of dried ionic liquid was placed on top of a freshly cleaved mica surface for experiment. Data were randomly sampled around the droplet where both the N 1s and Si 2p signals can be detected simultaneously. Here, data are plotted as a function of the Si 2p signal, which is an indicator for the layer thickness.

Our data demonstrates that (1) anions are enriched statistically, if clean mica is wet by a dry IL (here more then 50 XPS measurements were conducted). In contrast wetting of an ion exchanged surface with dry IL leads to a clear cation enrichment. In addition, the relatively strong chemical shift of the anion towards low binding energy in the anion rich and dry case strongly supports this argument (see SI of our previous work [4]). Second, (2) that anions are enriched at the vacuum interface in the dry wetting layer on mica (see again [4]) These two findings do not contradict each other. In contrast, we can improve and concretize the interpretation of Deyko et al. [3] as follows: The overall IL layer formed on dry mica contains statistically more anions, if wet by a dry compared to a wet bulk IL. At the same time the

outer layer indicates a relatively higher anion content. Following our argument that anions over adsorb in the inner layer, the second layer must be enriched by cations, while third layer will likely contain a higher numbers of anions. As a consequence, in XPS one will find a tendency to more anions in the outer layer if the inner layer is enriched by anions. But given the fact that the overall ratio at 0 degree detector angle (where the surface sensitivity for an incomplete 8 Å - 12 Å and 1-3 monolayers thick film at 3 nm attenuation length is negligible) statistically shows a higher anion count (see e.g. Figure S5) which strongly supports the conclusion that anions are enriched in the over all layer, and likely at both the mica/IL and the IL/vacuum interface.

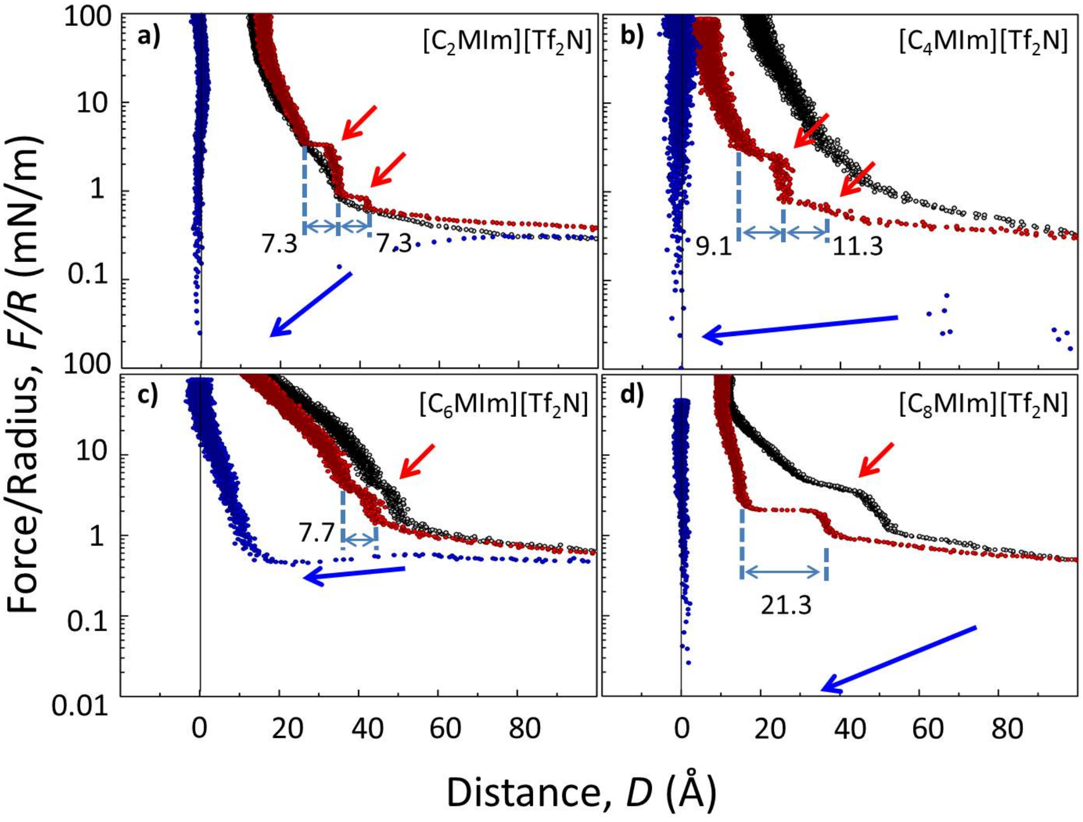

Figure S6. Full set of SFA data for series of ILs, including [C_2min][Tf_2N], [C_4min][Tf_2N], ,[C_6min][Tf_2N] and [C_8min][Tf_2N] in wet (blue), dry (black) and humid (red) environments, respectively.

References.

1. Li, K. and T. Kobayashi, *A Ft-Ir Spectroscopic Study of Ultrasound Effect on Aqueous Imidazole Based Ionic Liquids Having Different Counter Ions*. **Ultrasonics Sonochemistry**, 2016. 28 39-46.
2. Jiang, W., Y. Wang and G.A. Voth, *Molecular Dynamics Simulation of*

Nanostructural Organization in Ionic Liquid/Water Mixtures. **Journal of Physical Chemistry B**, 2007. 111(18) 4812-4818.

3. Deyko, A., T. Cremer, F. Rietzler, S. Perkin, L. Crowhurst, T. Welton, H.P. Steinruck and F. Maier, *Interfacial Behavior of Thin Ionic Liquid Films on Mica*. **Journal of Physical Chemistry C**, 2013. 117(10) 5101-5111.
4. Cheng, H.-W., P. Stock, B. Moeremans, T. Baimpos, X. Banquy, F.U. Renner and M. Valtiner, *Characterizing the Influence of Water on Charging and Layering at Electrified Ionic-Liquid/Solid Interfaces*. **Advanced Materials Interfaces**, 2015. 2(12).
